# Efficient Synapse Memory Structure for Reconfigurable Digital Neuromorphic Hardware

**DOI:** 10.3389/fnins.2018.00829

**Published:** 2018-11-20

**Authors:** Jinseok Kim, Jongeun Koo, Taesu Kim, Jae-Joon Kim

**Affiliations:** ^1^Department of Creative IT Engineering, Pohang University of Science and Technology, Pohang, South Korea; ^2^Department of Electrical Engineering, Pohang University of Science and Technology, Pohang, South Korea

**Keywords:** neuromorphic system, spiking neural network, spike-timing-dependent plasticity, on-chip learning, transposable memory

## Abstract

Spiking Neural Networks (SNNs) have high potential to process information efficiently with binary spikes and time delay information. Recently, dedicated SNN hardware accelerators with on-chip synapse memory array are gaining interest in overcoming the limitations of running software-based SNN in conventional Von Neumann machines. In this paper, we proposed an efficient synapse memory structure to reduce the amount of hardware resource usage while maintaining performance and network size. In the proposed design, synapse memory size can be reduced by applying presynaptic weight scaling. In addition, axonal/neuronal offsets are applied to implement multiple layers on a single memory array. Finally, a transposable memory addressing scheme is presented for faster operation of spike-timing-dependent plasticity (STDP) learning. We implemented a SNN ASIC chip based on the proposed scheme with 65 nm CMOS technology. Chip measurement results showed that the proposed design provided up to 200X speedup over CPU while consuming 53 mW at 100 MHz with the energy efficiency of 15.2 pJ/SOP.

## 1. Introduction

Spiking neural networks (SNNs) have received attention mostly due to their biological plausibility. It is known that biological neurons communicate with others by transmitting action potentials or spikes which represent dramatic changes in membrane potential (Maass, 1997; Gerstner et al., 2014). Although the performance of SNN such as accuracy in object recognition is lower than that of state-of-the-art deep neural networks (DNNs), SNNs are gaining interest because of their biological plausibility and unique characteristics such as time delay information and energy efficiency (Izhikevich, 2006; Du et al., 2015).

Artificial neural network (ANN) commonly uses massive number of computations (neurons) and parameters (synapses) in parallel, thereby causing a von Neumann bottleneck in conventional machines. Several hardware accelerators have been introduced to efficiently handle the necessary computations such as matrix multiplications. However, SNN contains time dimension in its processing and uses binary spikes for internal communication between neurons. Hence, it does not need enormous number of multiplications. These features cannot be utilized unless dedicated hardware is made. Therefore, even more dedicated hardware system is preferable for SNN (Seo et al., 2011; Arthur et al., 2012; Cassidy et al., 2013; Benjamin et al., 2014; Furber et al., 2014; Merolla et al., 2014; Akopyan et al., 2015; Davies et al., 2018; Frenkel et al., 2018).

Many existing neuromorphic hardware have been built using analog or mixed-signal devices so that electricity or charge of devices can directly model the functionality of neurons and synapses. However, it is not easy for analog devices to implement various neuronal functions and reconfigure parameters such as synaptic connections after the hardware is built. Furthermore, hardware-software correspondence is preferred when the hardware is used as a platform for theoretical or algorithmic study about SNNs because we can test the functionality and performance of the hardware even before it is built. Therefore, several fully digital neuromorphic hardware systems have been developed recently.

Digital neuromorphic chips such as IBM's TrueNorth (Merolla et al., 2014; Akopyan et al., 2015) and Intel's Loihi (Davies et al., 2018) include dedicated logic and memory to digitally simulate neurons and synapses. Although neurons with relatively complex operations may need a significant amount of computing logic, synapses that are several hundred to thousand times more than neurons require more hardware for memory than logic. Considering that neuromorphic hardware usually holds all synaptic weights on chip, it is important to implement efficient synapse memory to represent larger and more complex network.

Analog or mixed-signal neuromorphic hardware may utilize next-generation memory such as Resistive RAM (ReRAM) and memristor to enable simultaneous addition of multiple synaptic weights and efficient thresholding (Bichler et al., 2012; Querlioz et al., 2013). On the other hand, for digital implementation, static RAM (SRAM) is typically used as an on-chip memory for synaptic parameters. On-chip SRAM can be used like a typical buffer to store word-by-word. However, it can also be used as a 2D crossbar array to represent synapses that exist between neurons.

One of the most important features of SRAM crossbar arrays is that they can only be accessed in a row or column direction at a time. The direction of word line (WL) can be read at the same time. However, the direction of bit line (BL) cannot be read simultaneously. Row-wise access to synapse memory is preferred for higher throughput because inference operation which requires reading a row of synapses when a presynaptic spike arrives is more frequent and important. We can use column-wise memory access to read multiple synapses in a column at once and sum all synapses in a single cycle, but this requires much more complex hardware when the precision of synapses is high. In addition, using column-wise access in inference operation does not take advantage of input sparsity because it also reads synapses connected to presynaptic neurons without spikes. Park and Kim (2018) have suggested that using neuromorphic cores with different (row/column) directions can improve performance by reducing inter-core delay.

In spite of having higher throughput, SRAM crossbar arrays suffer from large area and power consumption. Merolla et al. (2014) have used crossbar architecture with column-wise access to the memory and multiple cycles to sum synapses that are read at once, thereby saving the area for the neuron module. Some existing hardware did not use crossbar architecture. Frenkel et al. (2018) have emulated the crossbar structure by using the time-multiplexed structure without using real crossbar array. Davies et al. (2018) have used routing tables to efficiently implement sparse and more complex synaptic connections.

Meanwhile, learning mechanism is very important in the study of SNN for machine learning applications or biological models. Spike-Timing-Dependent Plasticity (STDP) learning, the most popular learning method for SNN, uses difference between timing of a presynaptic spike and that of a postsynaptic spike. It potentiates synaptic connection when postsynaptic spike is emitted soon after the presynaptic spike is emitted (pre-then-post or causal case). It depresses it when presynaptic spike occurs after the postsynaptic spike is emitted (post-then-pre or acausal case).

The pre-then-post case requires the update of synapses connected to the same postsynaptic neuron while the post-then-pre case requires to update synapses connected to the same presynaptic neuron. In other words, updating the weights in the same row or same column is required when 2-D crossbar array is used to represent dense synaptic connections between axons and neurons. For memristive crossbar array, specific voltage pulse signals can be provided to the entire row and column (Querlioz et al., 2013). As the voltage across the memristive memory cell changes the state of the memory cell, it is possible to update any row/column of synaptic values simultaneously. However, for SRAM that is typically used in digital implementations, we can only access cells in either the row or column direction. Using one direction of memory access can severely degrade performance of either pre-then-post or post-then-pre STDP operation.

A possible solution to this problem is to modify the SRAM to enable bidirectional or transposable memory access. Seo et al. (2011) have designed custom 8-transistor (8T) SRAM with additional WL and BL for column-wise memory access. However, additional transistors and signal lines (WL and BL) will significantly increase the area of the memory cell and customized memory design is required.

The learning algorithm itself can be also modified instead. Pedroni et al. (2016) suggested to update synaptic weights when the presynaptic spike arrives and the presynaptic spike timer expires. However, with this algorithm, the exact timing can be inferred and used for the pre-then-post case when there is only one postsynaptic spike between two consecutive presynaptic spikes. Therefore, a large refractory period needs to be set to ensure such a condition. Davies et al. (2018) have used a similar approach called epoch-based synaptic modification. At an interval of several time steps, every presynaptic neuron's routing table is scanned to check spikes and update synapses. It also needs to assume sparse spikes for accurate learning. In addition, it needs to spend much time to scan all presynaptic neurons even if there is no postsynaptic spike. Although neither study used a crossbar array for synapse memory, the aforementioned learning algorithms can be directly applied to crossbar architecture.

In this paper, we proposed several schemes for the implementation of efficient synapse memory arrays to address these aforementioned issues.

By exploiting per-axon presynaptic weight scaling factors to synaptic weights, we reduced the total number of bits used for synapses while maintaining computation accuracy.

Using the offset parameters to define connections between axons and neurons, we can reduce the number of synapses from each axon and prevent wasting synapses when complicated network structure such as multi-layer network is implemented in the system. Furthermore, with our novel transposable memory addressing scheme, we can enable transposable access to the synapse memory using conventional 6T SRAM and make the throughput for learning with the same as the throughput for inference. Based on the schemes, we also demonstrated a 65 nm CMOS neuromorphic core chip optimized in terms of performance and area required for synapse memory.

## 2. Materials and methods

### 2.1. Spiking neural network (SNN)

In SNN, the membrane potential values of neurons can be described by various differential equations. Spikes from other neurons can directly modify the potential or change the amount of current through the membrane. The amount of difference in the potential is called Postsynaptic Potential (PSP). The leaky integrate-and-fire (LIF) model is one of the most basic models that considers the cell membrane as a capacitor with leakage current. The LIF model in discrete time domain can be expressed as

(1) $$\begin{array}{rc} V_{k}\left(t\right) & =V_{k}\left(t-1\right)-\alpha \left(V_{k}\left(t-1\right)-V_{\text{rest}}\right)+V_{\text{in}}, \end{array}$$

where *V*_*k*_(*t*) is a membrane potential of neuron *k* at time *t*, α is a leakage coefficient, *V*_rest_ is a resting potential, and *V*_in_ is an external input from outside or other neurons. If there is no disturbance, the potential is maintained at *V*_rest_. *I* is the summation of synaptic weights from presynaptic spiking neurons in the simplest case. When the membrane potential exceeds neuron's threshold, action potential occurs. After the occurrence of action potential, the neuron enters into a state during which it no longer integrates synaptic input.

### 2.2. Proposed hardware design

#### 2.2.1. System configuration

**Figure 1:**
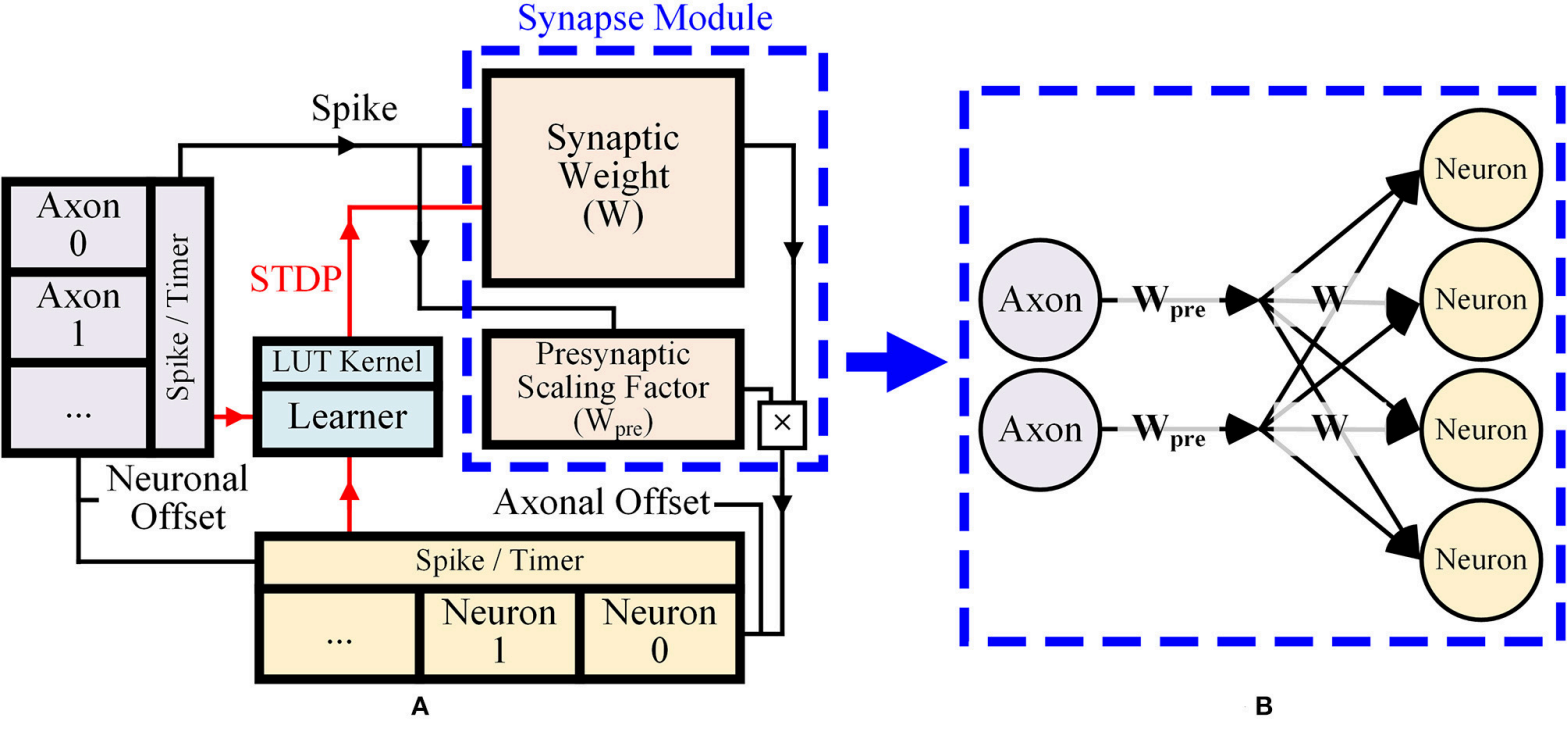
**(A)** Overall dataflow of the proposed design, **(B)** Presynaptic weight scaling scheme.

Figure 1A shows a simple description of the overall architecture and dataflow of the system. Executing one iteration of the inference operation and the learning operation composes a single time step that represents 1 ms in the simulation. In the inference stage, a spike from the axonal memory accesses the corresponding location of synapse memory. The amount of the synaptic weight value is sent to the neuronal memory for integration. To reduce the total number of memory bits for synapses, we proposed a presynaptic weight scaling scheme (Figure 1B). In the scheme, a presynaptic scaling factor of each axon is multiplied by original synaptic weights to obtain effective synaptic weights (Figure 1B). This concept will be explained more in detail in section 2.2.2. In the proposed system, membrane potential values of the neurons are changed as

(2) $$\begin{array}{r} V_{j}\left(t\right)=V_{j}\left(t-1\right)-\alpha \left(V_{j}\left(t-1\right)-V_{\text{rest}}\right)+\underset{i}{\sum }s_{t,i}\times w_{i}^{\text{pre}}\times w_{i,j}, \end{array}$$

where *i* is an index of the axon, *j* is an index of the neuron, *s*_*t, i*_ is a binary presynaptic spike from axon *i* at time *t*, *w*_*i, j*_ is a synaptic weight between axon *i* and neuron *j*, and $w_{i}^{\text{pre}}$ is a presynaptic scaling factor of axon *i*. Each neuron processes its membrane potential according to the Leaky Integrate-and-Fire (LIF) spike model and emits spikes back to axons.

The axonal and neuronal modules have the axonal and neuronal timers that count elapsed time steps from each axon and neuron's last spike. The axonal/neuronal timer values are incremented by one when one iteration of the inference operation and the learning operation is finished. When the maximum timer value (15) defined by their bit precision (4 bits) is reached, they no longer increase. The axonal timer value is set to zero when the axon sends out a presynaptic spike to neurons at the current time step, and the neuronal timer value is set to zero if the neuron fires.

**Figure 2:**
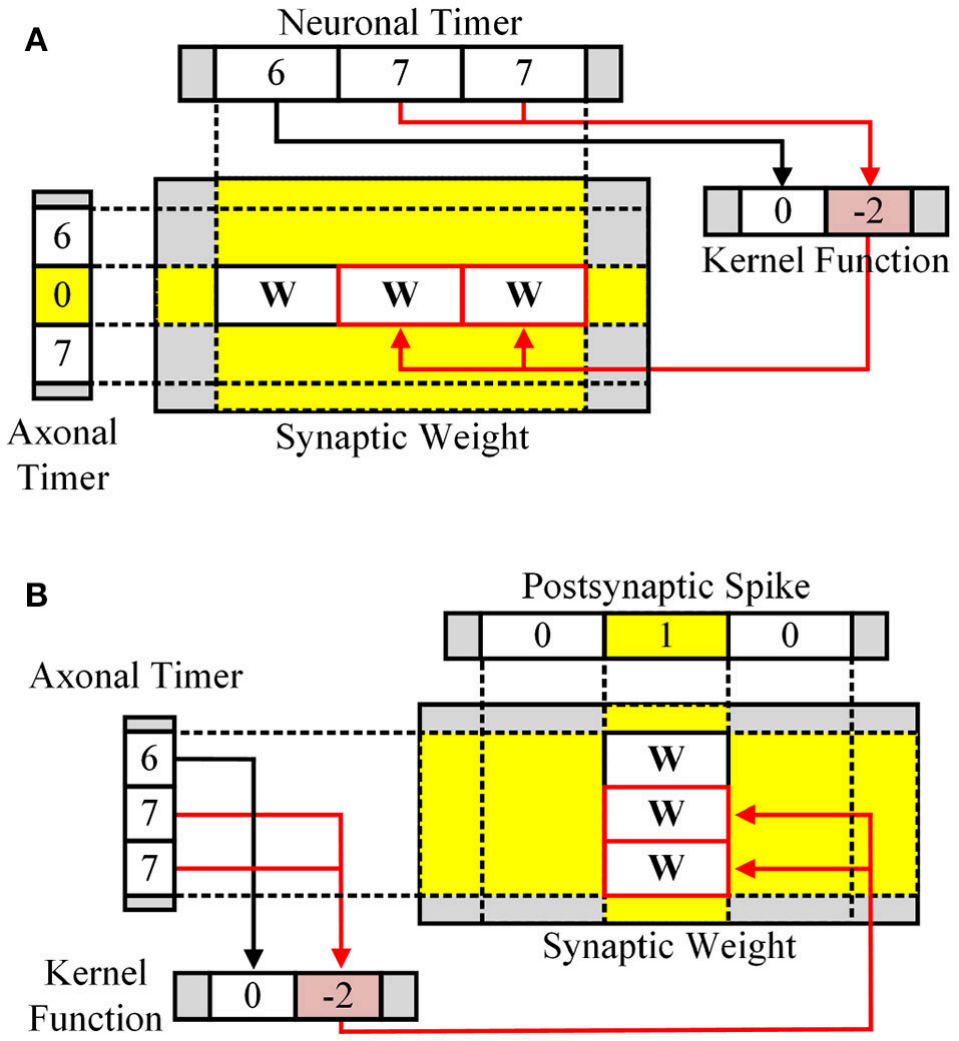
Learning stage for **(A)** pre-then-post spikes and **(B)** post-then-pre spikes.

In the learning stage, based on current spikes and the axonal/neuronal timers, the kernel function for STDP is applied to the synaptic memory using a lookup table (LUT). For post-then-pre spikes, the axonal timer is checked every time step (Figure 2A). The zero value at the axonal timer means the presynaptic spike has just occurred. Neuronal timer value at the moment represents the timing difference between the presynaptic spike and the postsynaptic spike. The neuronal timer value is used to select kernel value from the kernel function defined for each timing difference. Figure 2B shows the case for pre-then-post spikes, in which the axonal timer value represents the timing difference between the postsynaptic spike and the presynaptic spike, when the neuronal timer value is zero.

In our design, the synapse module is composed of presynaptic memory and synaptic memory. The system is designed to update only synaptic weights in the learning stage. The amount of synaptic weight change is obtained as kernel function value divided by the presynaptic scaling factor. The presynaptic scaling factors can be updated off-chip.

**Figure 3:**
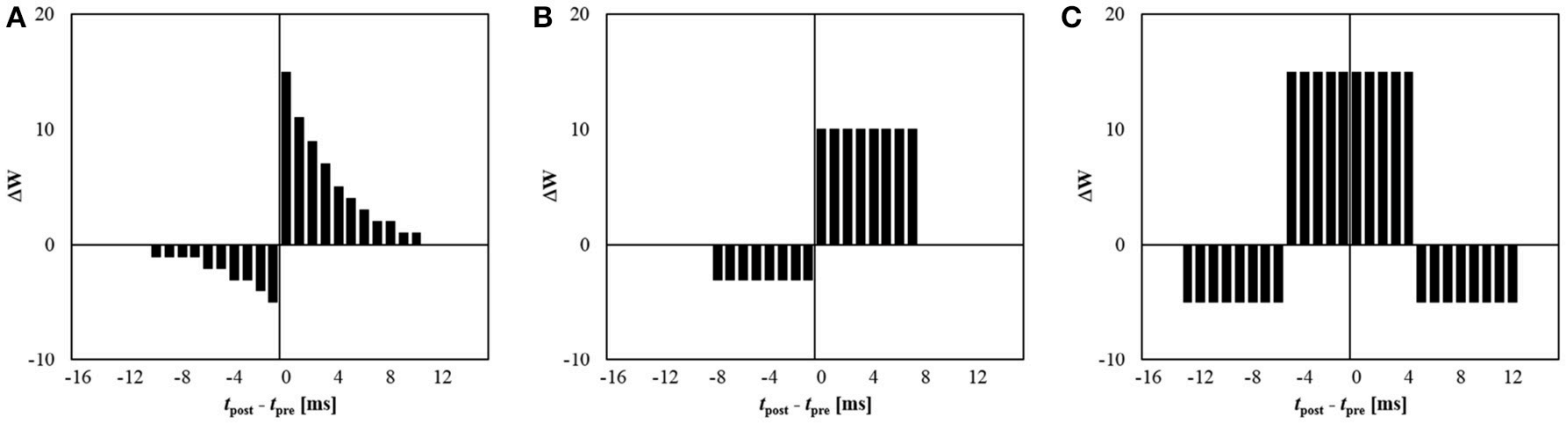
Examples of possible kernel functions for 5-bit synapse: **(A)** exponential STDP, **(B)** simplified STDP, and **(C)** symmetric STDP.

Meanwhile, by properly setting the kernel function, various learning rules can be easily implemented. Our system can hold eight different kernel functions at the same time, and each kernel function consists of 16 different signed values. Figure 3 shows some examples, including conventional STDP learning with an exponential kernel function (Figure 3A), its simplified version (Figure 3B), and symmetric STDP (Figure 3C).

#### 2.2.2. Presynaptic weight scaling

In the proposed system, a synaptic weight is multiplied by another value called a presynaptic scaling factor when it is used in the inference operation. Here we assume that the scaling factor depends on the presynaptic cell (axon) only while the ordinary synaptic weight depends on individual connection between the presynaptic cell and the postsynaptic cell (neuron).

**Figure 4:**
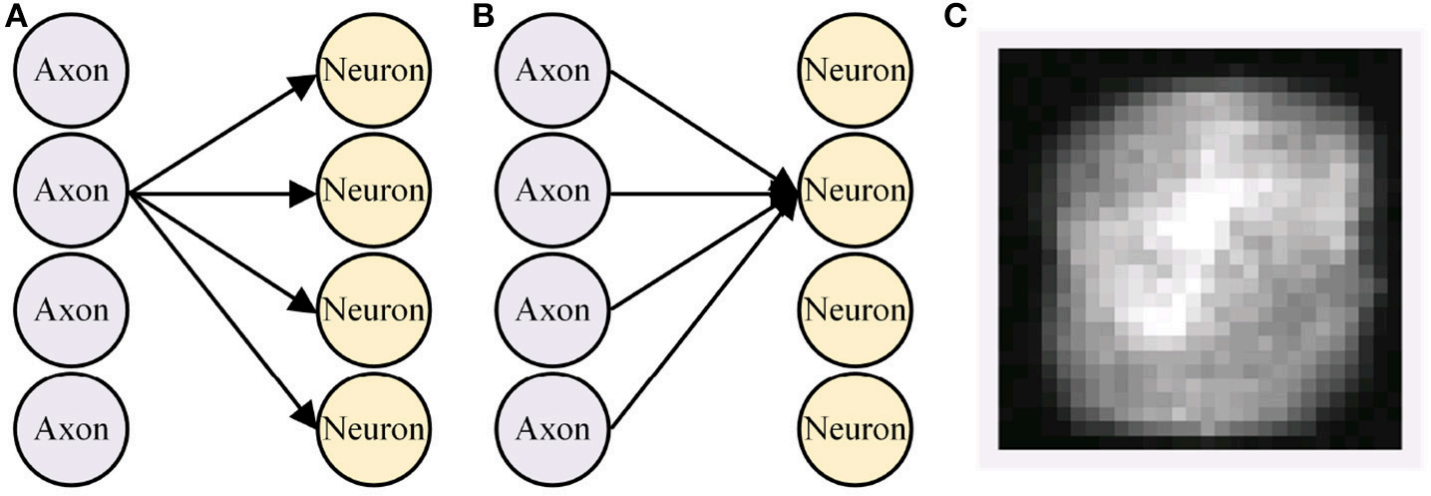
Definition of **(A)** pre-group and **(B)** post-group, and **(C)** average of each pre-group synapses' absolute values.

The presynaptic weight scaling scheme was adopted based on our observation that synapses from one axon might have more significant values than synapses from another axon, whereas synapses from an axon to different neurons have similar absolute values on average. As an example, we take 784 × 240 weights from a 784-240-10 multi-layer perceptron (MLP) that classifies MNIST handwritten digit dataset. A group of synapses from the same axon (Figure 4A, let us call this a pre-group) represents how much the axon contributes to each higher-level feature. A group of synapses that are connected to the same neuron (let us call this a post-group, Figure 4B), commonly called a kernel or a filter, tend to find distinctive patterns from pixels. Each pixel in Figure 4C represents the average of absolute values of 240 synaptic weights from each pre-group.

Visualization showed that weights from some axons tended to have higher absolute values than others. The average standard deviations (σ) of weights in a single pre-group/post-group was 0.0239/0.0291, meaning that weights in each group tended to spread similarly. However, σ of the average absolute weight values in a single pre-group/post-group was 0.0125/0.0024, meaning that each pre-group's average absolute weight value was distributed 5 times more broadly than each post-group's value. Similarly, σ of the root mean square (RMS) of weights in a single pre-group/post-group was 0.0174/0.0050, showing more than 3 times difference.

Using the scaling scheme, the number of memory bits for synapses is changed from (#_synapses_ × bits-per-synapse) to (#_synapses_ × bits-per-synapse + #_axons_ × bits-per-scaling-factor). However, because of a presynaptic scaling factor, we can use fewer bits for each synaptic weight while maintaining the accuracy of inference/learning. Therefore, the total number of memory bits for synapses can be reduced. Detailed results from experiments will be described in section 3.1. Note that, unlike conventional weight decomposition methods such as low rank approximation (Denton et al., 2014; Kim et al., 2016), 2D synaptic weight memory which keeps its size and dimension can provide flexibility and reconfigurability for on-line learning and manual modification of weights.

#### 2.2.3. Transposable memory addressing scheme

Assuming that the synaptic weight memory is 2D array and each row of it represents synapses from each axon to many neurons while each column of it represents synapses from many axons to each neuron, it only needs to be accessed row-wise in the inference stage. However, it must be accessed both row and column-wise in the learning stage because STDP learning uses both pre-then-post and post-then-pre spikes. Meanwhile, column-wise access to conventional SRAM-based memory requires row-by-row accesses because multiple cells attached to the same bit line cannot be read simultaneously. Such multiple row-wise accesses for one column-wise access is very wasteful in terms of both delay and energy. To address this issue, a custom transposable memory has been introduced (Seo et al., 2011). However, it requires non-standard customized memory design. In addition, it suffers from significantly increased cell area.

As an alternative, we propose a scheme to implement a transposable synapse memory using conventional 6T SRAM arrays. In this scheme, the memory is split into multiple blocks and data are remapped so that each memory block does not contain adjacent cells from the same column in the original memory. Then both row-wise and column-wise access can be done in a cycle by accessing divided memory blocks with different addresses.

Let us assume that the number of columns in the original weight matrix is *C* and the number of memory blocks is *B*. We rearrange elements of the matrix so element (*x*, *y*) of the original matrix (0 ≤ *x*, 0 ≤ *y* < *C*) goes to {(*x* + *y*)mod*B*}-th memory block with address (*Cx*+*y*)/*B*. Accessing *B* consecutive cells from element (*x*, *y*) in row direction requires elements (*x*, *y* + *b*) while accessing values in column direction requires elements (*x* + *b*, *y*) (for both cases 0 ≤ *b* < *B*). To access these values in *B* split memory blocks, an address we have to provide for *k*-th memory block (0 ≤ *k* < *B*) becomes

(3) $$\begin{array}{r} {\text{Addr}}_{k}=\begin{array}{c} \left\lfloor \frac{Cx+y+\left(\left(k-x-y\right)\text{mod}B\right)}{B}\right\rfloor \;\text{for row-wise access}\;\\ \left\lfloor \frac{C\left(x+\left(\left(k-x-y\right)\text{mod}B\right)\right)+y}{B}\right\rfloor \;\text{for column-wise access}\;\\ \; \end{array} \end{array}$$

Furthermore, *B* output values coming out from *B* blocks must be rearranged to provide final output values in the right order. It can be done by using a barrel shifter,

(4) $$\begin{array}{r} {\text{Output}}_{l}^{\text{final}}={\text{Output}}_{l+x+y\text{mod}B}^{\text{memory}}\text{for}0\leq l<B \end{array}$$

**Figure 5:**
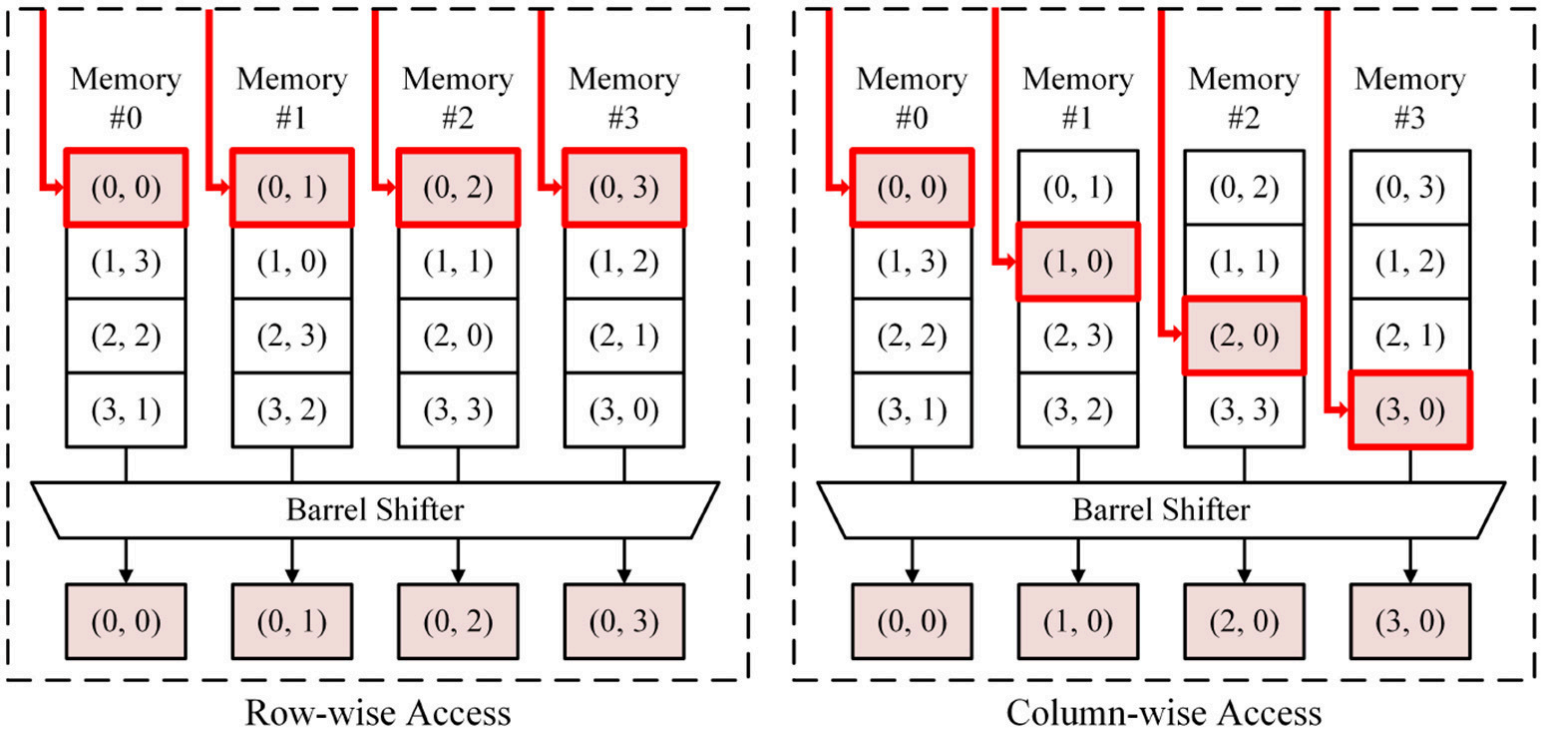
Transposable addressing scheme (*C* = 4, *B* = 4).

One example case is shown in Figure 5, in which *C* = 4 and *B* = 4. As shown in Figure 5, column-wise access can be done in a cycle by locating synaptic weights for the same neuron (the same *y*) in different blocks. Hence, inference and learning speed can be made the same with conventional 6T SRAM-based synapse memory. This addressing scheme can significantly increase the learning speed. Experimental results will be shown in section 3.2.

#### 2.2.4. Axonal/Neuronal offset

Conventional neuromorphic systems with 1K axons and 1K neurons are typically built with 1M synapses to support arbitrary synaptic connections between axon and neuron pairs. In contrast, only 256K synapses are used in our design for the same number of axons and neurons. As a result, the number of synaptic connections from an axon to neurons is reduced to 256 from 1K while the number of neurons remains at 1K. Let us define the number of synaptic connections from one axon to neurons as the axonal fan-out number (*N*_*f*_). Decreased *N*_*f*_ makes the number of columns in the synaptic weight memory become less than the number of neurons. Reducing the number of synaptic connections from each axon is obviously helpful in terms of memory reduction, but it needs to make sure that an axon can be connected to any neuron among 1K candidates.

**Figure 6:**
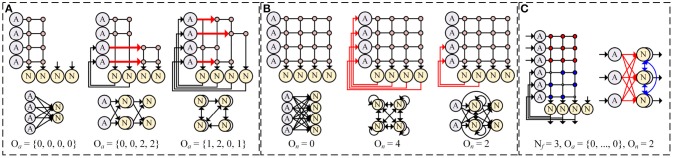
Network compositions with **(A)** different axonal offsets (*N*_*f*_ = 2) and **(B)** different neuronal offsets (*N*_*f*_ = 4), and **(C)** an example of implemented lateral inhibitory layer (*N*_*f*_ = 3).

As a solution, we introduce axonal offset *O*_*a*_ which makes synaptic weight *W*_*ij*_(*j* = 0, 1, …, *N*_*f*_) from axon *i* is connected to neuron *j* + *O*_*a*_(*i*). It allows an axon to reach any neurons with very small amount of additional resource for addressing. Figure 6A shows some examples of network compositions with different axonal offsets. A single layer (Figure 6A, left), a multi-layer (center), and a reservoir-like complex network (right) can be implemented with reduced number of synapses by changing axonal offsets. With axonal offset, the description of synaptic integration in the system is changed as

(5) $$\begin{array}{rc} V_{j}\left(t\right) & =V_{j}\left(t-1\right)+\underset{i\in I}{\sum }s_{t,i}\times w_{i}^{\text{pre}}\times w_{i,j-O_{a}\left(i\right)}^{\text{post}} \end{array}$$

(6) $$\begin{array}{rc} \text{I} & =\left\{i\in \mathbb{Z}\;|\;0\leq j-O_{a}\left(i\right)<N_{f}\right\}, \end{array}$$

where *i* is the index of the axon and *j* is the index of the neuron.

In addition, we use the neuronal offset (*O*_*n*_) to implement a complex network in a single core by allowing recurrent connections from neurons to axons (Figure 6B). As shown in the Figure 6B, the first *O*_*n*_ neurons have direct connections to the last *O*_*n*_ axons in sequence. It can be described as follows.

(7) $$\begin{array}{rc} s_{t+1,N_{a}-O_{n}+i} & =1\text{if}V_{i}\left(t\right)\geq \theta_{i},0\text{otherwise}\;\text{for}0\leq i<O_{n} \end{array}$$

where *N*_*a*_ is the number of axons, *i* is the index of the neuron, and θ_*i*_ is the threshold of neuron *i*.

With different *O*_*n*_, for example, synapse memory can be used to implement a single layer (Figure 6B, left), complete connections between every neuron and every axon for recurrent network (center), and intermediate choice to support both hierarchical and recurrent structures (right).

It is known that the accuracy of SNN strongly depends on the existence of a locally competitive layer commonly described as lateral inhibition (Oster and Liu, 2006). In a lateral inhibitory layer, a neuron which emits spikes immediately can prevent spikes in other neurons by inducing negative change in their membrane potentials. In our design, lateral inhibition can be implemented by using synapses with negative values and by making recurrent connections between neurons in the same layer with neuronal offset (Figure 6C).

In case the axonal fan-out number (*N*_*f*_) is smaller than the required number of synaptic connections from one axon in a specific network composition we want to implement, we can use multiple axons having different axonal offsets but receiving the same input. For example, if one axon is set to have the axonal offset of zero and another axon is set to have the offset of *N*_*f*_ (256), providing the same presynaptic spikes to those two axons conceptually enables synaptic connections from one axon to 2*N*_*f*_ (512) consecutive neurons.

In this case, we may have to allow one neuron to send one spike to multiple axons. However, our current design using the neuronal offset does not explicitly support this one-to-many neuron-to-axon connections. Instead, we can utilize known methods such as *splitter* (Merolla et al., 2014) or *neuron copy* (Esser et al., 2016). The *splitter* method uses one additional axon and multiple additional synapses/neurons so that the axon redirects received presynaptic spikes to those neurons. Then those neurons can send out the same spikes to different axons. The *neuron copy* method simply uses multiple neurons for the exact same neuronal states/functions so that they can produce identical postsynaptic spikes. Also note that this scalability issue will become a less burden when available hardware resources are increased by implementing multiple cores in the same chip as in Merolla et al. (2014) and Davies et al. (2018).

#### 2.2.5. Parameterized parallelization

**Figure 7:**
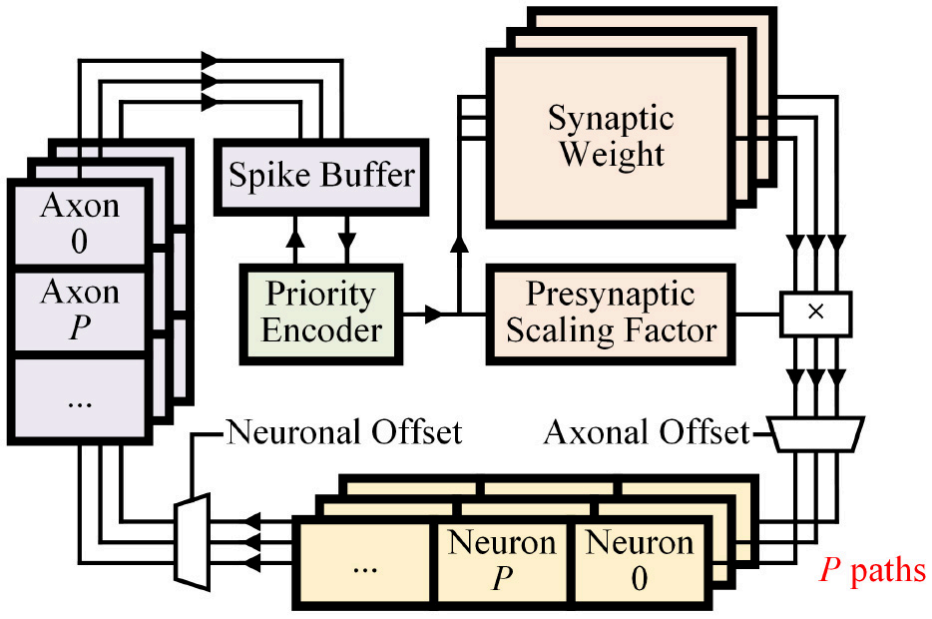
Dataflow of the design with parallelization number *P*. Learner module is omitted for simplicity.

For both inference and learning operations, parallelized memory access, and computation provide higher throughput. However, parallelization requires more execution units and resources. We propose parameterized parallelization to define the parallelization parameter *P* that controls the number of divided memory parts and execution units for the whole system (Figure 7). The architecture is designed to work with any *P*. Considering both performance and resource, we can conveniently compare designs with various *P*-values in field programmable gate array (FPGA) or simulation before deciding the final value for application-specific integrated circuit (ASIC) implementation.

The synaptic integration process can be parallelized by dividing synaptic memory and neuronal memories into *P* blocks (Figure 7). By dividing memories, *P* synaptic weights can be simultaneously read and delivered to *P* different neurons. Each separated neuronal memory block holds parameters of (#_Neurons_/*P*) neurons. Although *P* synapses are connected to different neurons, indices of neurons are consecutive. Thus, the connection can be made by a single *P* × *P* barrel shifter. Then *P* blocks of neuronal memories can independently perform the fire process and send spikes to *P* axons using a barrel shifter with neuronal offset.

*P* blocks of the axonal memory need to provide spikes one by one. Each presynaptic spike can then access the synaptic memory in the next time step. This parallelized integration process assures identical functionality for any *P*-value.

For transposable memory addressing, the number of split memory blocks (*B*) can be simply set equal to *P*. Then *P* synaptic weights can be read in one cycle in both inference/learning operations.

## 3. Results

We implemented an application-specific integrated circuit (ASIC) chip on a 65 nm CMOS process technology based on the proposed schemes. Before we fabricated the ASIC chip, we verified the effectiveness of the proposed schemes through software simulation and Field Programmable Gate Array (FPGA) implementation. We also used the flexibility of FPGA to investigate effects of various reconfigurable parameters to determine their optimal values for ASIC design.

### 3.1. Network accuracy

#### 3.1.1. Inference

**Figure 8:**
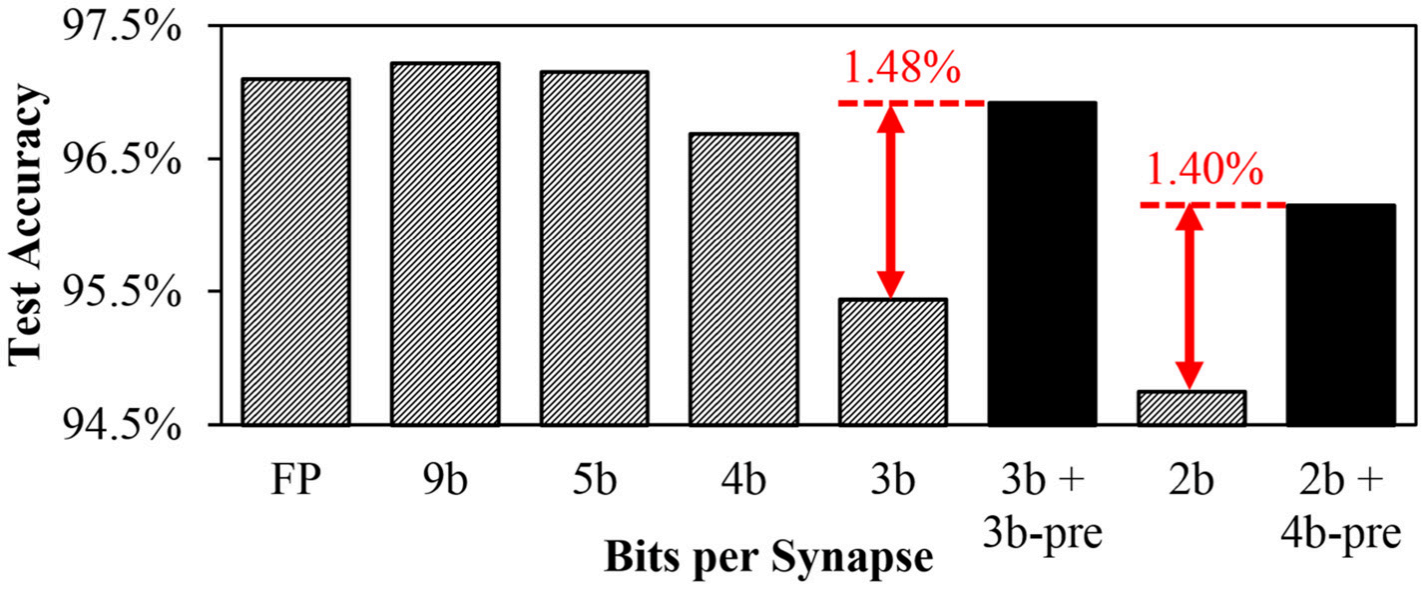
MNIST inference accuracy using different numbers of bits per synapse.

To see the impact of presynaptic weight scaling scheme on network accuracy, we tested different numbers of bits per synapse in MNIST digit recognition task. Using the method of Diehl et al. (2015), we built a rectified linear unit (ReLU) based network with size of 784-240-10 to classify MNIST dataset and trained it using back-propagation in MATLAB. After 15 epochs of training, we transferred trained weights into a SNN with the same number of neurons. Refractory periods and thresholds were empirically determined to be the same for all neurons. We then measured the inference accuracy for various number of bits per synapse (Figure 8). Inference results were determined by the number of spikes in the output neurons during 50 ms of simulation. Without any preprocessing on input images, Poisson spike trains with spike rates proportional to intensities were used as network inputs.

For a given number of bits per synapse *b*, synaptic weights were linearly quantized to 2^*b*^ different levels. The largest and the smallest quantization levels for each *b* were determined by grid search to provide the best accuracy.

As the first step to apply the proposed weight scaling, a presynaptic scaling factor value for each axon is determined by statistical methods, such as Root-Mean-Square (RMS) of weights from each axon or the average of absolute values of those weights. In this work, presynaptic scaling factors were set to be proportional to the RMS of the original synaptic weights connected to each axon. Note that presynaptic scaling factor value for each axon is also quantized based on the given bit resolution.

Figure 8 shows accuracy results from MNIST inference task using different numbers of bits per synapse. It was observed that 5-bit fixed-point weights provided similar accuracy to 32-bit floating point weights. The accuracy decreased as the number of weight bits decreased. While using 3-bit weights resulted in significant accuracy loss, the accuracy was substantially recovered if the weight scaling scheme was applied by adding 3-bit presynaptic scaling factor to the 3-bit synaptic weight (*3b + 3b-pre*). It can be observed that the accuracy of *3b + 3b-pre* case is even better than the 4-bit weight case. In addition, with the scaling scheme, 2-bit synaptic weights with 4-bit presynaptic scaling factors provided higher accuracy than 3-bit synapses. While the weight scaling scheme showed better accuracy, the amount of required synapse memory was reduced. For example, the memory requirement for *2b + 4b-pre* synapses is 4 × (784 + 240)+2 × (784 × 240+240 × 10) = 385, 216 bits in total. It is 33% smaller than that for 3-bit synapses which is 3 × (784 × 240 + 240 × 10) = 571, 680 bits. The *3b + 3b-pre* synapses require 25% less amount of memory than 4-bit synapses.

#### 3.1.2. STDP learning

Similarly, we also examined the impact of weight precision on STDP learning. We built a network with a single layer of 784–256 neurons and applied STDP while providing input spikes representing MNIST images. These 256 neurons have lateral inhibitory connections to each other for more effective unsupervised learning. The role of STDP in this example is to train synaptic weights so that each of 256 neurons becomes sensitive to a unique input pattern. At the same time, lateral inhibition prevents other neurons from becoming sensitive to the pattern. It is important to make sure that synapses connected to each neuron learn different patterns. On the other hand, these synapses should not be too responsive to a single image or a particular pixel.

From this perspective, using low-precision synaptic weights for STDP learning is difficult because the magnitude of the minimum weight change becomes relatively large and the sensitivity to a particular pattern increases rapidly, making it difficult to learn various patterns using thousands of different images. In contrast, synaptic weights with high numerical precision can gradually increase the sensitivity to specific patterns.

To maintain the amount of weight changes on average, weight updates must be applied in stochastic manner. By using STDP kernel value as the probability of weight change, relatively stable learning can be achieved with even extremely low-precision weights such as binary weights (Seo et al., 2011). In this case, the proposed weight scaling method helps more accurate STDP learning. In our scheme, the effective synaptic weight is obtained by the product of presynaptic scaling factor and synaptic weight. To keep the largest weight of the network the same with the presence of presynaptic scaling factor, the relative magnitude of original synaptic weight decreases, resulting in a smaller minimum weight change. In our hardware design, it is possible to increase neuronal threshold to effectively lower synaptic weights.

**Figure 9:**
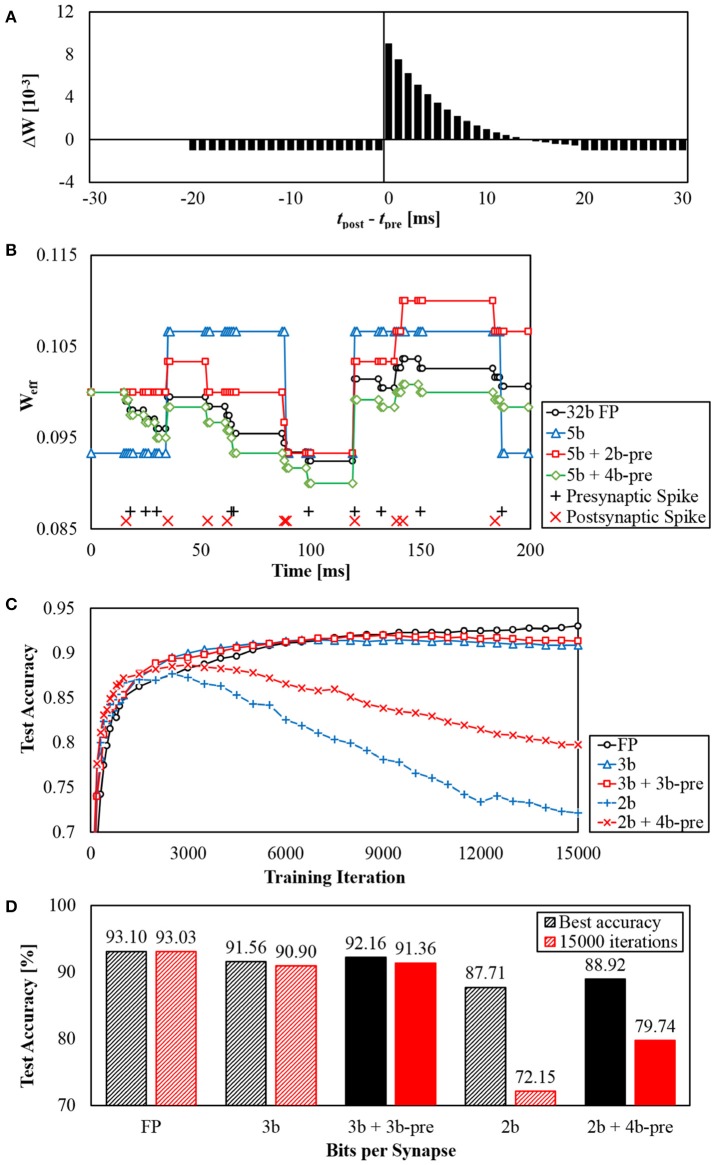
**(A)** STDP kernel function used, **(B)** Example of effective weights changed through STDP learning with different synaptic weight precision, and **(C,D)** MNIST accuracy results from using STDP + SVM classifier.

Figure 9 shows results from STDP learning with the scaling scheme. Using a customized STDP kernel function (Figure 9A), an example of changes in an effective weight during STDP is shown in Figure 9B. For given presynaptic spikes and postsynaptic spikes (+ and × signs at the bottom), a synaptic weight with 32-bit floating-point (FP) precision is changed as shown in the black line with circle markers. Using the weight with 5-bit precision (blue and triangle), possible weight values are restricted to few levels. With 2-bit presynaptic scaling factors, the minimum weight change is smaller up to four times (red and square). With 4-bit presynaptic scaling factors, the minimum weight change is smaller up to 16 times (green and diamond), thereby making weight changes much more similar to weight changes using floating-point precision.

To see its influence on accuracy as we used STDP as an unsupervised learning method, we added and trained a linear Support Vector Machine (SVM) classifier after the STDP layer as in Kheradpisheh et al. (2018). At each training iteration, one training image was shown to the network. At every 100 iterations, we trained a new SVM classifier using only images that we had used to train STDP layer. We did not restrict the precision of parameters in SVM classifier to only see the effectiveness of STDP layer. Similar to section 3.1.1, input spikes were given as Poisson spike trains during STDP while only 30 ms was given for each image to prevent over-fitting.

As a result, for different cases of synaptic precision, we were able to get test accuracy results as 0–15,000 training iterations are processed (Figure 9C). In floating-point (FP) case, the best accuracy was achieved at 15000 iterations and the accuracy kept increasing. Cases with 3-bit synapse also showed similar results, but the accuracy reaches the highest point before 15,000 iterations. However, the accuracy of 2-bit synapses began to degrade significantly at about 3,000 iterations. Lower synaptic precision leads to larger minimum weight change, making learning more unstable and hard to converge. Therefore, adding presynaptic scaling factors to scale down the minimum weight change can reduce accuracy degradation due to this unconvergence.

Figure 9D shows the best accuracy results achieved by each case until 15,000 iterations and the accuracy after 15,000 iterations. The presence of presynaptic scaling factors slightly increased the best accuracy for both 3-bit and 2-bit synapses. The difference became noticeable for accuracy at 15,000 iterations.

### 3.2. Performance

**Figure 10:**
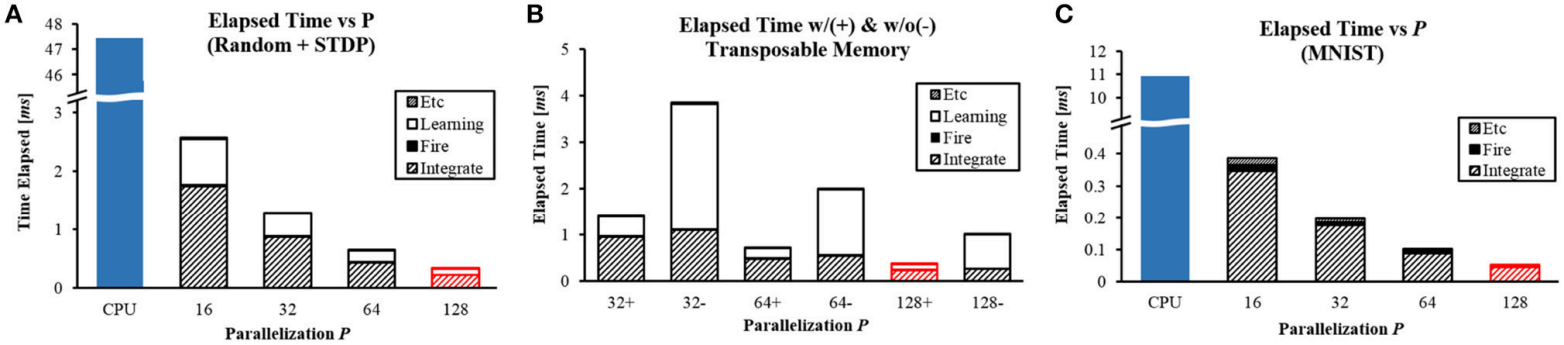
Computation time with different *P* values for **(A)** random network with STDP learning, **(B)** with (+) and without (−) the transposable addressing scheme, and **(C)** MNIST inference task.

To compare the performance of the system with conventional machines, we wrote an optimized software program. It works exactly the same way as the hardware implementation does in C++. Intel Xeon E5-2609 v3 (1.90 GHz clock) was used to measure CPU performance. To see the difference caused by changing the parallelization parameter *P* as explained in section 2.2.5, we measured computation time in FPGA implementation of our design (Figure 10). The result from *P* = 128 was identical to our chip measurement result (red figure). The design was synthesized and placed at clock frequency of 100 MHz.

To fully utilize these implemented features, we made two example cases for the experiment: one with parameters that were randomly set throughout the system (Figures 10A,B) while another from a real application (Figure 10C). For both cases, total numbers of axons/neurons were 1K/1K and *N*_*f*_ = 256. The number of bits for a presynaptic scaling factor was fixed at 4-bit and that for a synaptic weight was fixed at 5-bit.

The first example network was a random network made of 5-layer of 256-256-256-256-256 neurons. STDP using a simple exponential kernel function was applied to the second layer. An average spike rate of neurons was 54.74 Hz. The speed increased in proportion to *P*. At *P* = 128, it provided **130**× speedup against CPU (0.364 ms vs 47.43 ms, Figure 10A).

The inference stage consists of two sub-stages: integrate and fire. The execution time of the sub-stages could be overlapped to some degree by pipelining, but integrate and fire stages often use the same memory and execution units. Therefore, we did not pipeline them for simpler control logic. Computation time can be divided into three different components: learning, integrate, and fire. In Figure 10A, most of computation time is consumed for integration while learning also takes a considerable amount of time. Note that time taken for learning is reduced significantly by the proposed transposable addressing scheme. We synthesized another design without using the proposed addressing scheme to compare the performance between designs with and without the transposable access scheme (Figure 10B). For three different *P* values, the proposed transposable addressing scheme provided **6.55**× speedup in the learning stage and 2.75 × speedup in the total delay on average.

As the second example network, we pre-trained 784-240-10 MLP to classify MNIST dataset and converted those parameters into a SNN for the experiment based on the scheme proposed by Diehl et al. (2015). Simulation was done for 30 ms for each image. The inference speed for one image is shown in Figure 10C. The speedup increased up to **205**× when *P* = 128 because of its smaller spike rate (8.71 Hz on average) compared to the first example.

**Figure 11:**
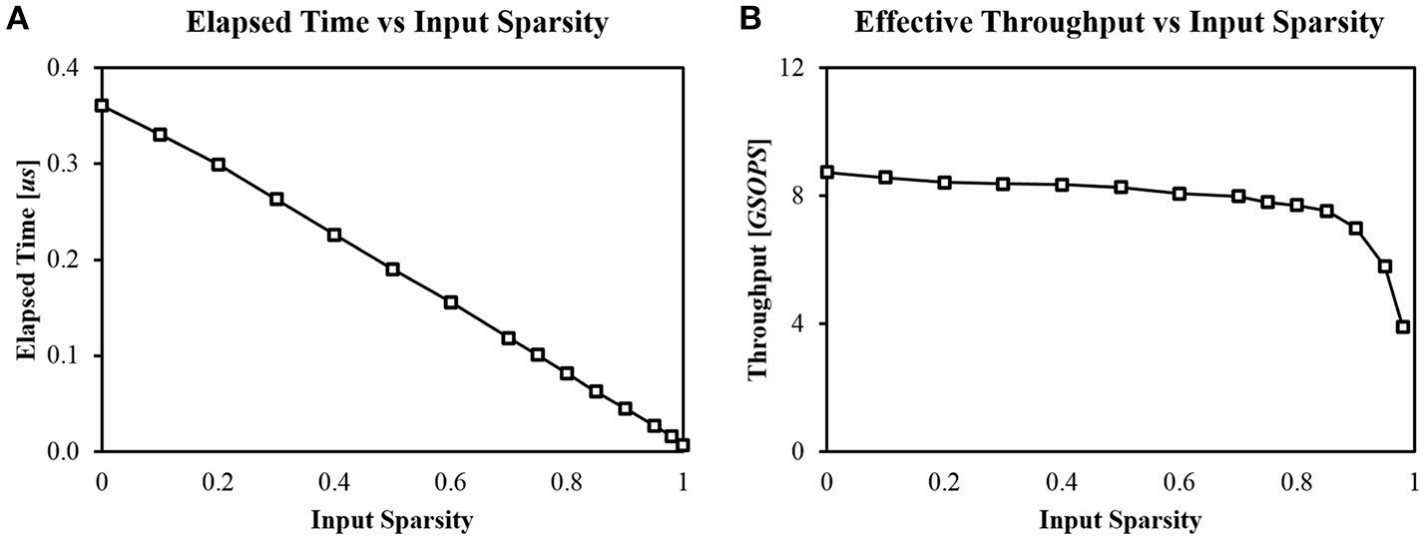
**(A)** Computation time with different input sparsities (*P* = 128, 1024 × 256 random network) and **(B)** Corresponding effective throughput of the system.

Another key feature of our design is its event-driven nature enabled by the priority encoder and the spike buffer (Figure 7). Zero inputs can be easily skipped by checking *P* axons in one cycle. Therefore, regardless of input sparsity, the system can provide high throughput constantly. We measured computation time with different input sparsity, using a single layer (1, 024 × 256) SNN with random weight values without learning operation. *P* was fixed to 128. Computation time was linearly proportional to the amount of non-zero input (Figure 11A).

Using the measured computation time, we can calculate effective throughput of the system. Ideally, our system can provide a maximum of 12.8 GSOPS (Giga Synaptic Operations Per Second) when *P* = 128 at 100 MHz clock because it can process *P* synaptic operations by reading *P* synapses in a single cycle. The weight sparsity is assumed to be zero in this case. However, not every cycle is used for synaptic integration. The most reliable way to calculate effective throughput is to divide the number of total synaptic operations by the total processing time (Figure 11B). The throughput of the system remains almost constant until the input sparsity becomes too high (>95%). The maximum throughput was **8.73 GSOPS** when the input sparsity is zero.

### 3.3. Resource utilization

Although larger *P* provides significant speedup, it requires more execution units running at the same time. We measured resource utilization in FPGA implementation to compare different options using Xilinx ZC706 evaluation board with Zynq-7000 XC7Z045. There are three different measures of the resource: look-up table (LUT), flip-flop (FF), and block random access memory (BRAM). ZC706 board has a total of 218,600 slice LUTs, 437,200 FFs, and 545 36 Kb BRAMs. LUTs are mostly used for logic. FFs are mostly used for registers while BRAMs are used for memories.

**Figure 12:**
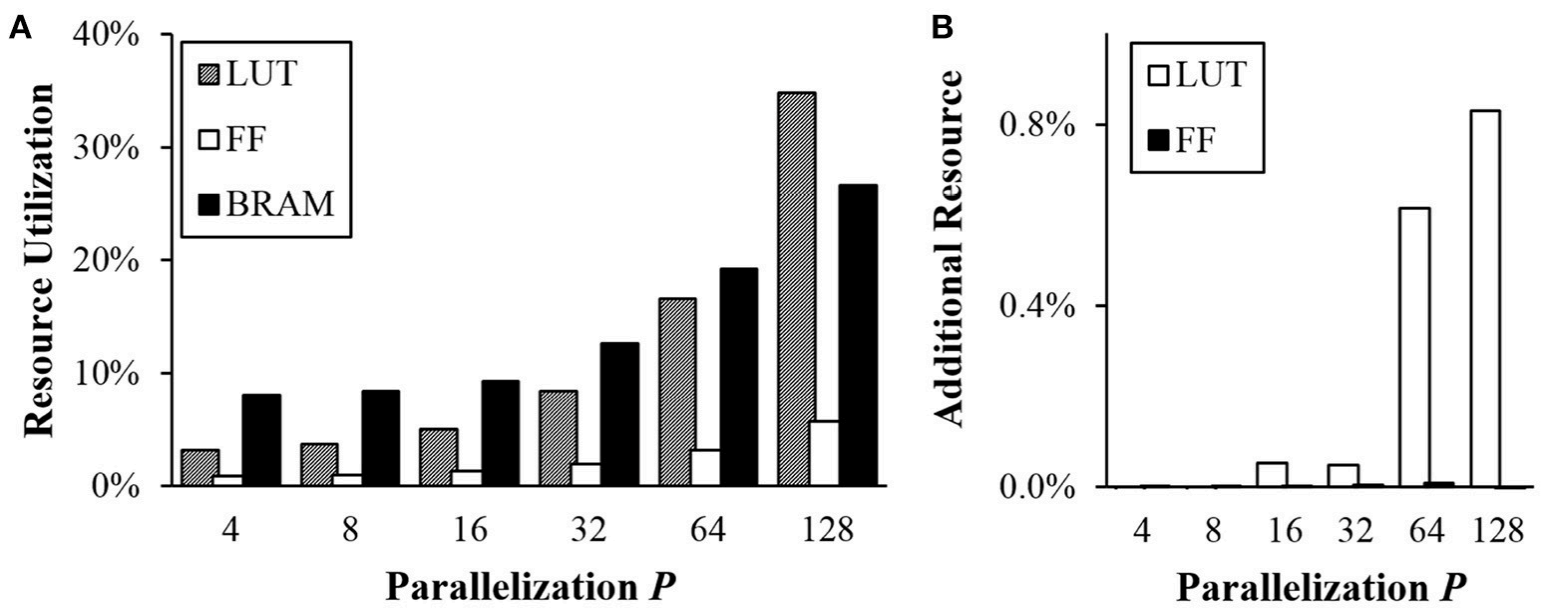
Resource utilization in FPGA **(A)** vs. *P* and **(B)** overhead for the transposable addressing scheme.

Figure 12A shows relative resource utilization in FPGA for different *P*-values. Utilization for both LUTs and FFs increased proportionally to *P*. When *P* = 128, 34.79% of LUTs and 5.72% of FFs were used. Utilization for BRAM also increased when *P* ≥ 32 because each instance of divided memories takes at least a half BRAM block due to I/O issue. Note that total bits of memory used are not changed when *P* changes.

As explained in section 2.2.3, the proposed transposable access to the synaptic memory requires additional hardware resource to calculate addresses and rearrange output values. Additional resource utilization for the proposed scheme was also measured (Figure 12B). Note that only small amounts (< 1%) of additional LUTs are needed when *P* is larger than 8.

### 3.4. Power consumption and area

Based on the proposed schemes and experimental studies, we fabricated a neuromorphic core using a 65 nm CMOS process technology. The number of neurons is 1K and the number of synapses is 256K. *P* was set to 128. The number of bits per presynaptic scaling factor/synaptic weight was set to 4b/5b.

**Figure 13:**
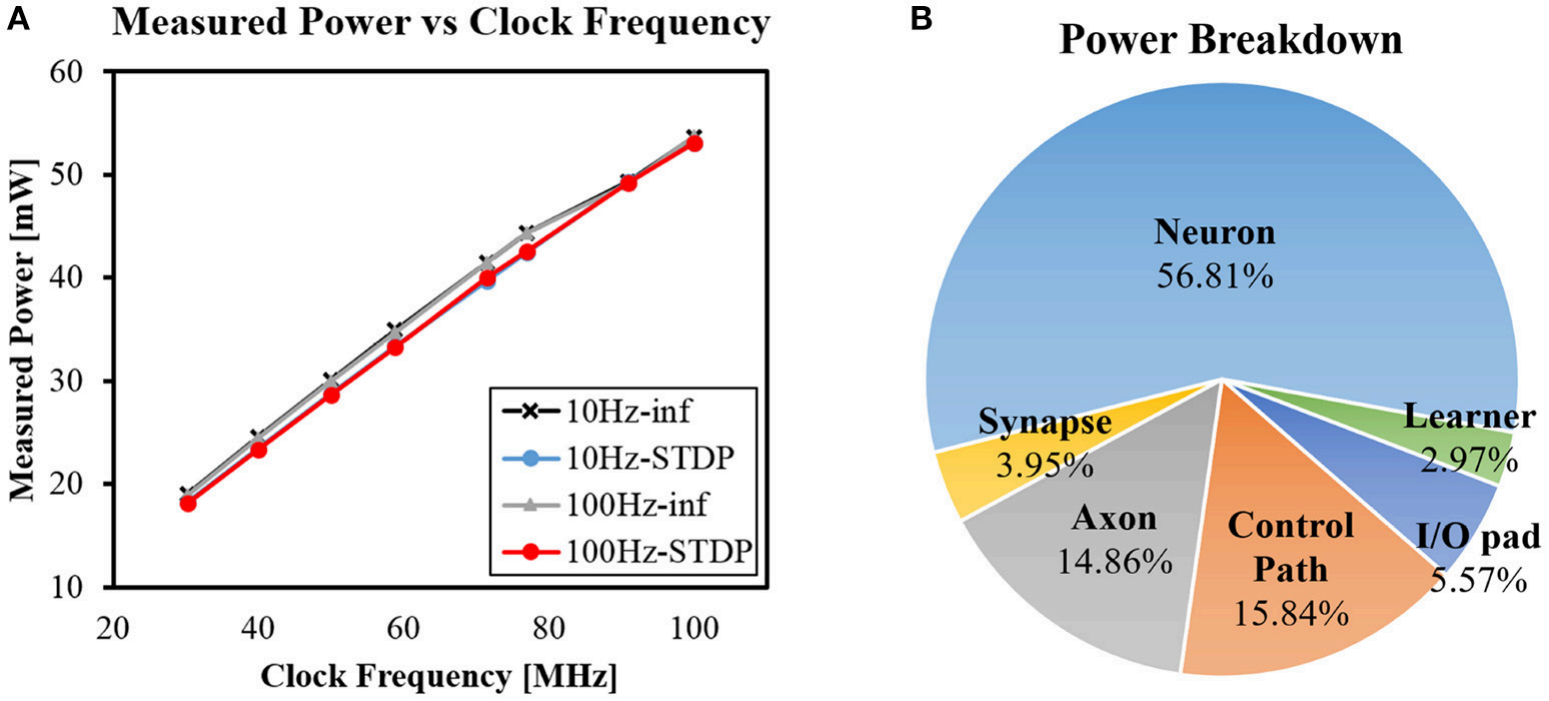
**(A)** Measured power with different clock frequencies and **(B)** Power breakdown by functional module.

Figure 13A shows chip power measurement results at 1.2V operating voltage. The power was measured while running the network with STDP and lateral inhibition which was described in section 3.1.2. We measured four different cases by changing maximum input spike rate (10/100 Hz) and turning on/off STDP learning (STDP/inf). For all four cases, the power consumption increased linearly as clock frequency increased from 30 to 100 MHz. At clock frequency of 100 MHz, the chip consumed 53.10/53.07 mW for inference only and 53.70/53.58 mW for STDP learning with 100/10 Hz input spike rate. Note that neither higher input spike rate nor the existence of learning stage made a noticeable difference in power consumption. More computations due to more spikes or the learning stage are handled by increased computing cycles as explained in section 3.2. The throughput for accessing the synapse memory in the learning stage becomes the same as the throughput in the inference stage by using the proposed transposable memory access.

Note that the energy consumption for the learning operation is still larger than that for the inference operation due to the larger number of cycles.

The power efficiency of our chip is **65.9 GSOPS/W** and the energy efficiency is **15.2 pJ/SOP**. The power/energy efficiency was calculated using the power measurement results and the effective throughput. In section 3.2, the effective throughput of the system was calculated using performance measurement data (Figure 11B). When the input sparsity is 0.9 (90%), the effective throughput was 6.99 GSOPS. In case the weight sparsity is 0.5 (50%) as described in Akopyan et al. (2015), the effective throughput is reduced to 3.50 GSOPS.

Power breakdown by functional module was also obtained by post-place and route power estimation using PrimeTime PX (Figure 13B). More than half of the total power is consumed in neuron module. Note that power consumption for synaptic integration is mostly counted as neuron module since neuronal values are updated using synaptic values.

**Figure 14:**
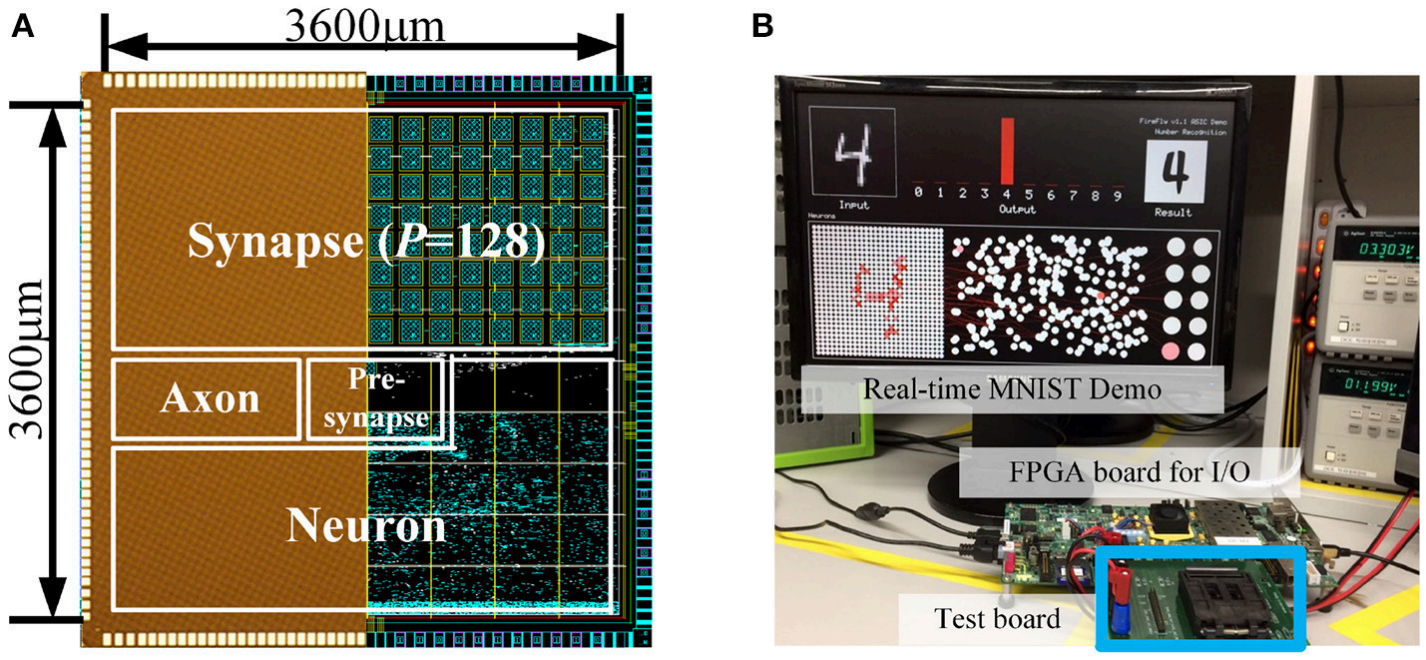
**(A)** Die microphotograph and layout of the design (65 nm CMOS) and **(B)** Demo & measurement environment.

The layout and the die photo of the design are shown in Figure 14A. Total core area was 12.9 mm^2^. Figure 14B shows real-time demo and measurement environment (Supplementary Video).

## 4. Discussion

### 4.1. Comparison with other neuromorphic systems

Due to different design goals and approaches, it is not straightforward to quantitatively evaluate performance or efficiency of different neuromorphic hardware systems. Nevertheless, let us compare the energy efficiency and the throughput between our design and some well-known neuromorphic systems (Table 1). TrueNorth (Merolla et al., 2014; Akopyan et al., 2015) consists of 4,096 neurosynaptic cores with total 1 million neurons and 256 million synapses using 28 nm CMOS process. Loihi (Davies et al., 2018) was designed for more reconfigurability using on-chip x86 cores and 128 neuromorphic cores with total 128 thousands neurons and 2.1 million synapses in 14 nm CMOS process. Frenkel et al. (2018) implemented a neuromorphic core with 256 neurons and 64 thousands synapses in 28 nm CMOS process. Reported energy efficiencies of those systems are 26 pJ/SOP (TrueNorth), 23.7 pJ/SOP (Loihi), and 9.8 pJ/SOP^1^ (Frenkel's), respectively.

**Table 1:** Comparison with other neuromorphic hardware systems in terms of energy efficiency and throughput.

**Hardware**	**Process**	**Energy efficiency [pJ/SOP]**	**Throughput [GSOPS]**
TrueNorth (Akopyan et al., 2015)	28 nm CMOS	26	58
Loihi (Davies et al., 2018)	14 nm CMOS	23.7	N/A
Frenkel et al., 2018	28 nm CMOS	9.8	0.035
Ours	65 nm CMOS	15.2	8.73

The energy efficiency of our chip (15.2 pJ/SOP) seems better than that of TrueNorth and Loihi, but we do not intend to claim that our system has better energy efficiency than TrueNorth and Loihi because of different synaptic precisions and neuronal functions. Especially, both TrueNorth and Loihi have more neurons and larger networks so the required hardware for routing is much more complicated and inevitably consumes more energy.

One of the main focuses in our design was to have high throughput using the parallelized dataflow. The maximum throughput of our design is 8.73 GSOPS when the input/weight sparsity is 0/0. The throughput of our design for typical operation condition (input/weight sparsity = 0.9/0.5) is 3.5GSOPS. In comparison, TrueNorth reported the throughput of 58 GSOPS (Akopyan et al., 2015), which can be translated to 14 MSOPS per each neurosynaptic core. However, each core in TrueNorth spends significant amount of time for routing spikes to other cores through Network-on-Chip (NoC). If the time for processing spikes inside the core is considered only, then the maximum throughput of each TrueNorth core is increased up to 988 MSOPS with 1 GHz local clock. With input/weight sparsity 0.9/0.5, it is reduced down to 49.4 MSOPS.

The main reason for the difference in throughput between TrueNorth and our design is that our design explicitly parallelizes the neuronal module into *P* blocks (section 2.2.5) for larger throughput. In contrast, in a single TrueNorth core, only one synaptic weight is read and used for integration at each cycle. In addition, the reason why there is larger difference between the throughput of the two designs for higher input sparsity case is that our design has zero input skipping capability in the axon module while TrueNorth does not.

Loihi did not explicitly report measured/estimated throughput, but since it does not exploit crossbar memory to store sparse synaptic connections more efficiently, it may not be easy to handle many synaptic weights in a single cycle. Frenkel et al. (2018) also did not implement an actual crossbar array for synaptic connections so that the design also reads and uses only one synaptic weight at each cycle, which results in relatively low throughput (35 MSOPS) at 100 MHz clock frequency.

### 4.2. Necessity and overhead of barrel shifter

Because of the parallelized dataflow as explained in section 2.2.5, multiple (*P*) synaptic weights are read simultaneously for synaptic integration. The membrane potential values of *P* neurons are then updated at once and the addresses of neurons being updated are determined by the sum of column indices of synaptic weights and the axonal offset. Since column indices of weights are consecutive, the addresses of these updated neurons are also consecutive. To handle these neurons in a single cycle, each of *P* divided neuronal memory blocks holds values of a non-overlapping group of *N*_*n*_/*P* neurons (*N*_*n*_: total number of neurons in the system). In this case, if addresses of neurons covered by each memory block are different by *P*, then any consecutive *P* neurons can be processed by using all memory blocks at the same time (i.e., the first block holds values for the neuron 0, *P*, 2*P*, …, *N*_*n*_−*P*).

If the axonal offset is zero or a multiple of *P*, the first synaptic weight is always used for the first neuron memory block. In that case, each synapse weight only needs to have signal lines connected to one of the *P* neuron memory blocks. However, the axonal offset value may not be a multiple of *P*, especially when we want to use one neuromorphic core to configure various network compositions. For example, if the axonal offset is one, the first synaptic weight is used to update the neuron in the second neuron memory block. Therefore, depending on the axonal offset, *P* synaptic weights must be circularly shifted so that a *P* × *P* barrel shifter is used to deal with this issue.

**Figure 15:**
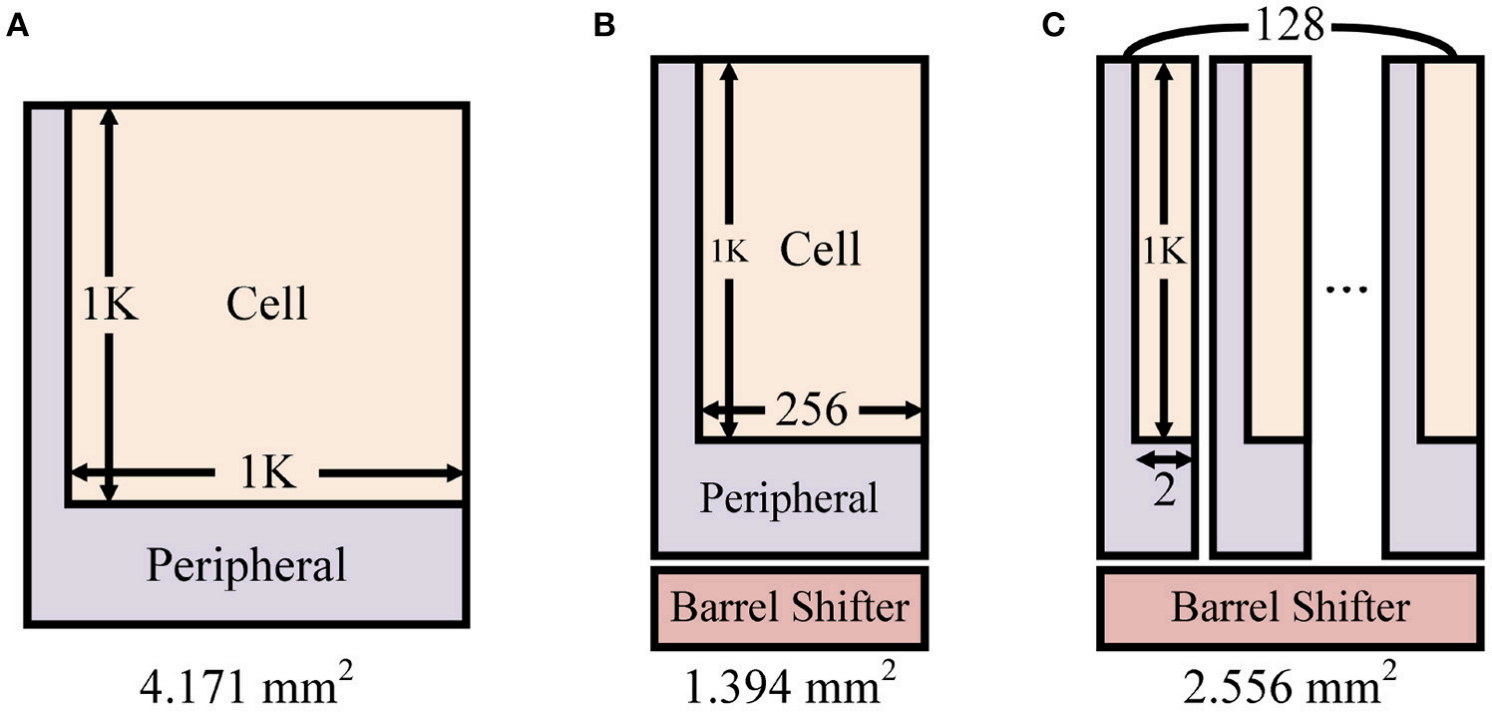
Different SRAM crossbar synapse memory design scenarios for 1K axons/neurons: **(A)** 1K × 1K crossbar, **(B)** 1K × 256 crossbar with a barrel shifter for the axonal offset concept, and **(C)** Split memory blocks (128 blocks) of 1K × 256 crossbar for the transposable addressing scheme.

To precisely analyze overhead of adding barrel shifter, we analyzed different design scenarios of synapse memory for 1K axons/neurons (Figure 15). As explained in section 2.2.4, the axonal offset exists to reduce the total memory size for synapse while enabling flexible synaptic connections between neurons in hardware. Without the axonal offset, 1K × 1K crossbar array is required for 1K axons/neurons (Figure 15A). We designed 1K × 1K memory using a memory compiler and measured its area (SRAM cells + peripheral components). To be more specific, because SRAM macro configuration such as maximum word size is constrained by the technology library, we designed 8 different crossbars each having 80-bit (5-bit synapse × 16) word size and 8,192 word lines. By doing so, 128 different synapses can be read/written at once. The memory area for this case was 4.191 mm^2^.

By reducing the crossbar size to 1K × 256 (Figure 15B, 8 crossbars with 80-bit word size and 2,048 word lines), the area of memory was reduced down to 1.225 mm^2^. For the axonal offset concept, a 128 × 128 barrel shifter is required and its area is 0.169 mm^2^. Then total area becomes 1.394 mm^2^. It means that the barrel shifter occupies only 12.1% of the total area. In all, by applying the axonal offset concept we can reduce the memory size by **3.01**× (4.191 vs. 1.394).

On the other hand, in the proposed transposable memory addressing scheme (section 2.2.3), the index of the memory block in which the first element exists is changed depending on the direction of memory access (row/column) and the address which needs to be accessed. Synaptic values read from multiple memory blocks must be rearranged or circularly shifted using a barrel shifter. Note that the axonal offset concept and the transposable addressing scheme require the same *P*×*P* barrel shifter between the synapse memory and the neuron memory for different reasons. Hence, the existing barrel shifter can be used for transposable memory addressing without extra overhead. Figure 12B shows that the amount of additional resource required for transposable addressing is negligible when a barrel shifter already exists for the axonal offset concept.

### 4.3. Additional overhead for transposable memory

In addition to the overhead of the barrel shifter, splitting the synaptic memory into multiple blocks for the transposable addressing may incur an additional overhead.

Split memory blocks are allowed to turn on word lines for different rows. Thus, it works as if an additional word line is added in diagonal direction. Existing word line can be used to access the row direction, and conceptually additional one can be used to access data diagonally. No additional transistor or signal lines are needed inside a memory block.

However, physical area of the design may increase because every split memory block uses its own peripheral circuits. Without the transposable access, using 1K × 256 crossbar array with the barrel shifter (Figure 15B) required 1.394 mm^2^. Splitting this crossbar into 128 different blocks (Figure 15C, 128 crossbars with 5-bit word size and 2,048 word lines) does not increase the area for SRAM bit-cells, but the increase of area due to the duplicated peripheral components in each block is not negligible. The total memory area including the barrel shifter increases up to 2.556 mm^2^, which is 1.83 × larger than original memory. We further improved it by designing semi-custom SRAM array that removes redundant peripheral circuits from each memory block without hurting the functionality, which resulted in only 1.20 × larger area compared to ordinary SRAM array. Note that using custom 8T SRAM for transposable access is usually known to require >2 × area. In our investigation with the cell layout, the custom 8T SRAM bit-cell based on Seo et al. (2011) was 2.48 × larger than common 6T SRAM bit-cell.

### 4.4. Weight decomposition in neural network

To reduce the memory size for parameters or the number of computations, many studies have proposed the idea to decompose a single weight value into a multiplication of two or more values (Denton et al., 2014; Kim et al., 2016). The biggest difference between existing methods and our scaling scheme is that ours does not reduces the number of parameters. In common approaches including low-rank approximation, a *M*×*N* matrix is decomposed into a multiplication of a *M*×*K* matrix and a *K*×*N* matrix (*M, N*>*K*) to reduce the total number of parameters. However, our presynaptic scaling uses a *M*×1 matrix and a *M*×*N* matrix. Thus, the number of parameters is slightly increased. Instead, we lowered the precision of values to reduce the total number of memory bits.

Since the conventional decomposition method determines a single synapse weight as the sum of multiplications of multiple values, it is difficult to modify some weights using on-chip learning algorithms such as STDP. Our scaling method also obtains a single weight value by multiplying multiple values, but only two values are used for each weight. Thus, it is relatively easy to modify specific weight values. Changing a single synaptic weight affects only one effective synaptic weight whereas in conventional decomposition methods changing a value in the decomposed matrix affects the whole row or column of weights.

### 4.5. Overhead for weight scaling

The proposed weight scaling scheme reduces the total number of memory bits significantly as described in section 3.1.1. However, the scheme incurs one additional burden. To obtain the effective synaptic weight, the presynaptic weight scaling factor and the synaptic weight must be multiplied. This multiplication was not needed without the scaling scheme, and moreover SNN hardware generally does not require any multiplication.

However, overhead for this multiplication is not significant. It is known that the power consumption for fixed-point multiplication is an order of magnitude smaller than that for SRAM memory access (Horowitz, 2014). Furthermore, the bit precision of presynaptic scaling factor/synaptic weight is only 4b/5b in our system, which is much smaller than common cases of 8-bit fixed-point numbers so that power consumption for multiplication is even smaller.

In terms of area overhead, without the scaling scheme, a single accumulator that accumulates 5-bit synapses into 16-bit potentials consumes 221 μm^2^ whereas a single multiply-and-accumulate that multiplies 4-bit and 5-bit weights and accumulates consumes 492 μm^2^. This clearly shows that the multiplication requires additional components, but the area overhead is negligible when compared to the area of a single synapse memory block, which is 18,402 μm^2^. Note that the required numbers of accumulators and the memory blocks in our system are exactly the same as *P*.

## 5. Conclusion

In this work, we designed an optimized SNN hardware accelerator architecture exploiting efficient synapse memory structure. The proposed presynaptic weight scaling provides 1.4% improvement in MNIST test accuracy using almost the same number of memory bits for synapse.

By reducing the bit precision of synapses, the scaling scheme achieved ~30% reduction in the number of memory bits while providing the same accuracy result.

Neuronal/axonal offset parameters were proposed for reconfigurabiltiy of network composition. Transposable addressing scheme for divided memory blocks was also exploited for 6 × faster STDP learning. Lastly, to consider performance/resource trade-off, we proposed parameterized parallelization scheme in the architecture. When fabricated in 65 nm CMOS process, ASIC implementation of our design provided 200 × speedup over general CPU in MNIST inference task while consuming 53 mW with the energy efficiency of 15.2 pJ/SOP.

## Author contributions

JiK, JoK, and J-JK designed hardware architecture. JiK, JoK, and TK conducted experiments on hardware and software. All of the authors contributed to the writing of manuscript.

### Conflict of interest statement

The authors declare that the research was conducted in the absence of any commercial or financial relationships that could be construed as a potential conflict of interest.

## Footnotes

^1^the exact condition of power measurement (ex. input/weight sparsity) is not known.
